# Endoscopic Image-guided treatment of Upper Gastrointestinal foreign body and nursing care of complications

**DOI:** 10.12669/pjms.37.6-WIT.4858

**Published:** 2021

**Authors:** Na Liu

**Affiliations:** 1 Na Liu, Attending Physician, Endoscopy Room, Heping Hospital Affiliated to Changzhi Medical College of Shanxi Province, Changzhi, 046000, Shanxi Province, China

**Keywords:** Endoscopy, Epidemiology, Foreign body in upper digestive tract, Nursing intervention

## Abstract

**Objective:**

This study used phased array imaging algorithm to explore the epidemiological characteristics of endoscopic treatment of upper gastrointestinal foreign bodies to provide a basis for nursing intervention.

**Methods:**

We collected data on the age, sex, cause, type of foreign body, success rate of removal, retention location, time and complications of patients with foreign bodies in the upper gastrointestinal tract who were treated in the emergency department of the Digestive Endoscopy Center in our hospital. The study was conducted from January 2018 to December 2020 and we also performed statistical analysis.

**Results:**

The high incidence of foreign bodies in the upper digestive tract was in 45 years old to 74 years old patients. The foreign body types were mostly food balls and sharp foreign bodies, accounting for 37.0% and 44.2%, respectively. The cause was misuse and the most accounted for 52.1%, followed by oesophageal pathological stenosis which accounted for 45.5%. The oesophagus in the retention site accounted for up to 80.0%, and the success rate of foreign body extraction was 96.4%. The complications of patients with foreign body retention within twenty four our retention were mainly esophageal scratches and traumatic esophagitis, accounting for 48.5%. 39.6%.

**Conclusion:**

There are high risks in the treatment of foreign bodies in the upper digestive tract. Targeted, prospective, and streamlined nursing interventions can provide patients with fast and professional medical care services and minimize patient pain.

## INTRODUCTION

Clinically, foreign bodies in the upper gastrointestinal tract are a common emergency, arising from foreign bodies or food clumps incarcerated in the upper gastrointestinal tract.^1^,^2^ Patients often feel pain or difficulty in swallowing.^3^,^4^ Common foreign bodies include fishbone and date nucleus. If not treated in time, it will cause secondary infection of the esophageal wall,^5^,^6^ such as abscesses and empyema, and septic shock may occur in severe cases.^7^,^8^

Endoscopic treatment is the primary treatment for foreign bodies in the upper digestive tract.^9^ Compared with traditional surgery, endoscopic treatment has many advantages, such as less trauma, fewer complications, quick recovery of patients, and lower treatment costs.^10^,^11^

In order to study the safety and effectiveness of endoscopic treatment of upper gastrointestinal foreign body. We analyzed the clinical characteristics of endoscopic treatment of upper gastrointestinal foreign body cases, and summarize the experience of endoscopic treatment.^12^.

## METHODS

This study included one hundred eighty two patients admitted in our hospital from January 1, 2018 to December 31, 2020, with suspected upper gastrointestinal foreign body. The age, sex, cause, type of foreign body, success rate of removal, retention location, time and complications of the upper gastrointestinal foreign body patients treated by emergency treatment in the gastrointestinal endoscopy center of our hospital were noted and analyzed after IRB approval (dated March 20, 2021) were analyzed statistically

### Inclusion criteria

1. Thoracic and abdomen lateral radiographs and CT scans or gastroscopes were diagnosed as patients with foreign bodies in the upper gastrointestinal tract; 2. Patients who were excluded from foreign bodies in the throat by a five-featured doctor. In one hundred sixty five cases, 156 were removed under awake state and 9 under general anaesthesia.

Data was collected through ***s***elf-designed upper gastrointestinal foreign body epidemiology questionnaire It included Gastroscopy reports of patients with upper gastrointestinal foreign body. Information collected included the patient’s age, gender, cause, total foreign body category, retention site, time and take-out success rate.

The patient takes the left side lying position, uses a matching oral guard, and inserts a gastroscope as usual. After finding foreign bodies, carefully observe the foreign body morphology, size, relationship with the lumen, and whether there are complications. Use mechanical principles and anatomical knowledge to choose appropriate equipment to withdraw foreign body together with gastroscope. CT and other examinations to assist in the assessment of the condition, close observation of changes in vital signs after surgery, proton pump inhibitor and mucosal protective agent should be used according to the situation, if necessary, fasting, gastrointestinal decompression, anti-infection, nasal feeding, enteral nutrition and other treatments should be considered.

### Statistical analysis

**Fig.1:**
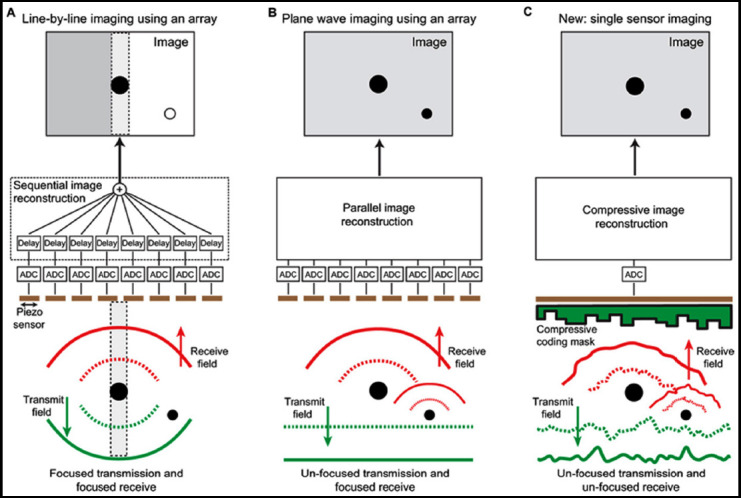
Geometric representation of delay time calculation.

Statistical methods such as χ^2^ test and correlation analysis are used. The first step is to use a single transducer element to transmit ultrasonic waves and make all the elements of the transducer array receive ultrasonic echoes. The geometric description of the calculation of the delay time in the sound field from the transmission to the reception of ultrasonic waves is shown in Fig.1.

The delay time of each array element is expressed as:


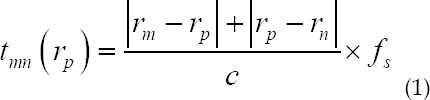


Where n and m represent the serial numbers of the transmitting and receiving array elements, respectively, N and M are the number of transmitting and receiving array elements, which is equal to the number of array elements of the entire array, n = 1, 2, .., N; m = 1, 2, .., M. C is the speed of sound of ultrasonic waves, *f_s_* is the sampling frequency of the system, *r_m_* and *r_n_* represent the spatial position of the transmitting array element and the receiving array element, and *r_p_* is the spatial position of the imaging point. The calculation process of LRI is


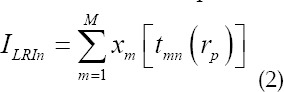


In the formula, *x_m_(t)* represents the echo data of m array elements. N LRI images are formed into HRI images by weighted superposition as


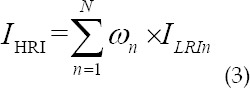


**Fig.2:**
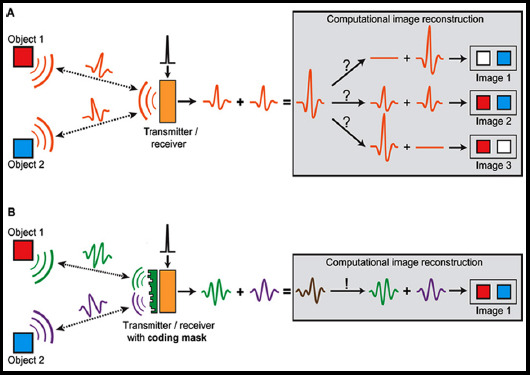
SA algorithm principle.

Where *ω_n_* is the weight function of the transmitting array element n. The principle of SA algorithm is shown in Fig.2.

In an ultrasound endoscopic imaging system, the Barker coded excitation process can be described as the convolution of the carrier pulse with the oversampled signal coded by Barker:


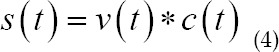


Where is the convolution, *v(t)* is the carrier pulse signal, and *c(t)* is the oversampling signal encoded by Barker, which can be expressed as


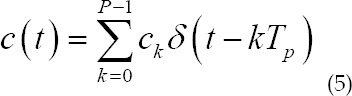


*C={c_k_* =±1,*k*=0,1,L, *P*-1} is the Barker code sequence, P is the Barker code length, for example, the 4-bit Barker code sequence is {1,1,1-1}, which is *T_p_* the unit chip time of Barker code. Thus, the time width of the Barker coded signal is *T = PT_p_*. The mathematical representation of the LMF carrier is 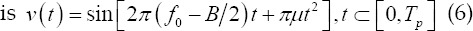


Where *f_0_* is the center frequency of the LFM carrier; μ is the bandwidth of the LFM carrier; *μ* is the FM speed, *μ=B/T_p_*.

## RESULTS

Of the 165-emergency upper gastrointestinal foreign body patients, 110 were male (66.7%) and 55 were female (33.3%). The male to female ratio was 2: 1, aged 4 to 81 years (43.5 years ± 15.7 years. According to the International Statistical Classification of Health Problems, the age division standard is divided into 45-54 years old, 55-64 years old, 65-74 years old, the incidence is higher, accounting for 18.8%, 23.0%, 26.7 % In this age group, the occurrence of oesophageal foreign bodies is more common due to pathological changes in the oesophagus and decreased dietary management ability. Secondly, the elderly patients have a higher risk of foreign body in the upper digestive tract due to the decreased ability to take care of themselves. The gender and age distribution of patients with foreign bodies in the upper gastrointestinal tract in emergency department are shown in Table-I.

**Table-I:** Gender and age distribution of patients with foreign bodies in upper gastrointestinal tract in emergency department.

Gender	No. of cases	4-14	15-24	25-34	35-44	45-54	55-64	65-74	75-81
Male	110	7 (6.4)	3 (2.7)	9 (8.2)	15 (13.6)	20 (18.2)	26 (23.6)	28 (25.5)	2 (1.8)
Female	55	1 (1.8)	3 (5.5)	5 (9.1)	7 (12.7)	11 (20.0)	12 (21.8)	16 (29.1)	0 (0.0)

One hundred sixty five cases of upper gastrointestinal foreign body types included food balls, button batteries, rings, coins, stomach stones and other blunt foreign bodies 76 cases; fish bones, bone spurs, pins, pharmaceutical aluminium foil, dentures, hairpins, blades, steel needles and other sharp foreign bodies There were eight cases of long foreign objects such as toothbrushes, pen sets, pencils, keys, etc.; 5 cases of complex foreign objects such as lighters, watches, nail clippers; three cases of oesophageal metal stent slipping. Among them, the majority are food groups, accounting for 37.0%. The types and locations of foreign bodies in patients with foreign bodies in the upper digestive tract are shown in Table-II.

**Table-II:** Successful removal and distribution of foreign bodies in patients with foreign bodies in upper gastrointestinal tract (n = 165).

*Foreign body type*	*No. of cases*	*Successfully removed*	*Part*				

			*Upper oesophagus*	*Mid-oesophagus*	*Lower oesophagus*	*Stomach*	*Duodenum*
Food group	61	61 (100.0)	27 (44.3)	23 (37.7)	11 (18.0)	0 (0.0)	0 (0.0)
Blunt foreign body	15	15 (100.0)	3 (20.0)	2 (13.3)	2 (13.3)	8 (53.3)	0 (0.0)
Sharp foreign body	73	69 (94.5)	28 (38.4)	10 (13.7)	25 (34.2)	7 (9.6)	3 (4.1)
Elongated foreign body	8	7 (7/8)	0 (0.0)	0 (0.0)	0 (0.0)	6 (6/8)	2 (2/8)
Complex foreign body	5	4 (4/5)	0 (0.0)	0 (0.0)	0 (0.0)	3 (3/5)	2 (2/5)
Oesophageal stent	3	3 (3/3)	0 (0.0)	0 (0.0)	1 (1/3)	2 (2/3)	0 (0.0)
Total	165	159 (96.4)	58 (35.2)	35 (21.2)	39 (23.6)	26 (15.8)	7 (4.2)

## DISCUSSION

In this study, CT scan or gastroscope was used to detect patients, with appropriate Barker code sequence and convolution pulse signal selected. Endoscopic imaging found that, the echo signal ratio was improved, and the signal-to-noise ratio also increases, raising the accuracy of diagnosis and treatment.

Some foreign bodies can pass spontaneously, while some cannot. Gezer et al.^13^ studied 1,000 children who accidentally ingested button batteries, and found that button batteries would pass spontaneously in 85% patients. Hong et al.^14^ researched 194 cases of foreign bodies in the digestive tract, and found 26.9% of the patients had complications, and 4 patients had to undergo surgery because the foreign body could not be removed. If the time exceeded 12 hours, endoscopy should be taken as soon as possible. In this study, the success rate of foreign body removal was 96.4%, and esophageal stricture accounted for 45.5%. The complications within 24 hours were mainly esophageal scratches and traumatic esophagitis, accounting for 48.5% and 39.6%, respectively.^15^,^16^

In terms of the care for patients with foreign bodies in the gastrointestinal tract, elderly patients were unwilling to go to hospital, and children have difficulty cooperating. In such cases, the medical staff should inform them about the necessity of the treatment and the specific treatment method. In this study, the subjects were all elderly patients who have a high risk during the treatment of foreign bodies in the digestive tract. Nursing interventions, which are targeted, forward-looking, and streamlined, can provide patients with fast and professional medical care services and minimize their suffering.

## CONCLUSION

Endoscopic treatment is the primary treatment method for foreign bodies in the upper digestive tract, which has good safety and effectiveness. While treating foreign bodies, it is also important to detect potential digestive tract diseases, treatment of primary disease or complications. With the continuous development of digestive endoscopy technology, some patients who originally required surgical treatment can achieve endoscopic treatment.
